# Supplementary material for: Neonatal gene therapy achieves sustained disease rescue of maple syrup urine disease in mice
Source: Nat Commun. 2022 Jun 7;13:3278. doi: 10.1038/s41467-022-30880-w (PMC9174284; doi:10.1038/s41467-022-30880-w)
Supplement: Supplementary file 1 — Supplementary Information [file 41467_2022_30880_MOESM1_ESM.pdf]

Figure S1

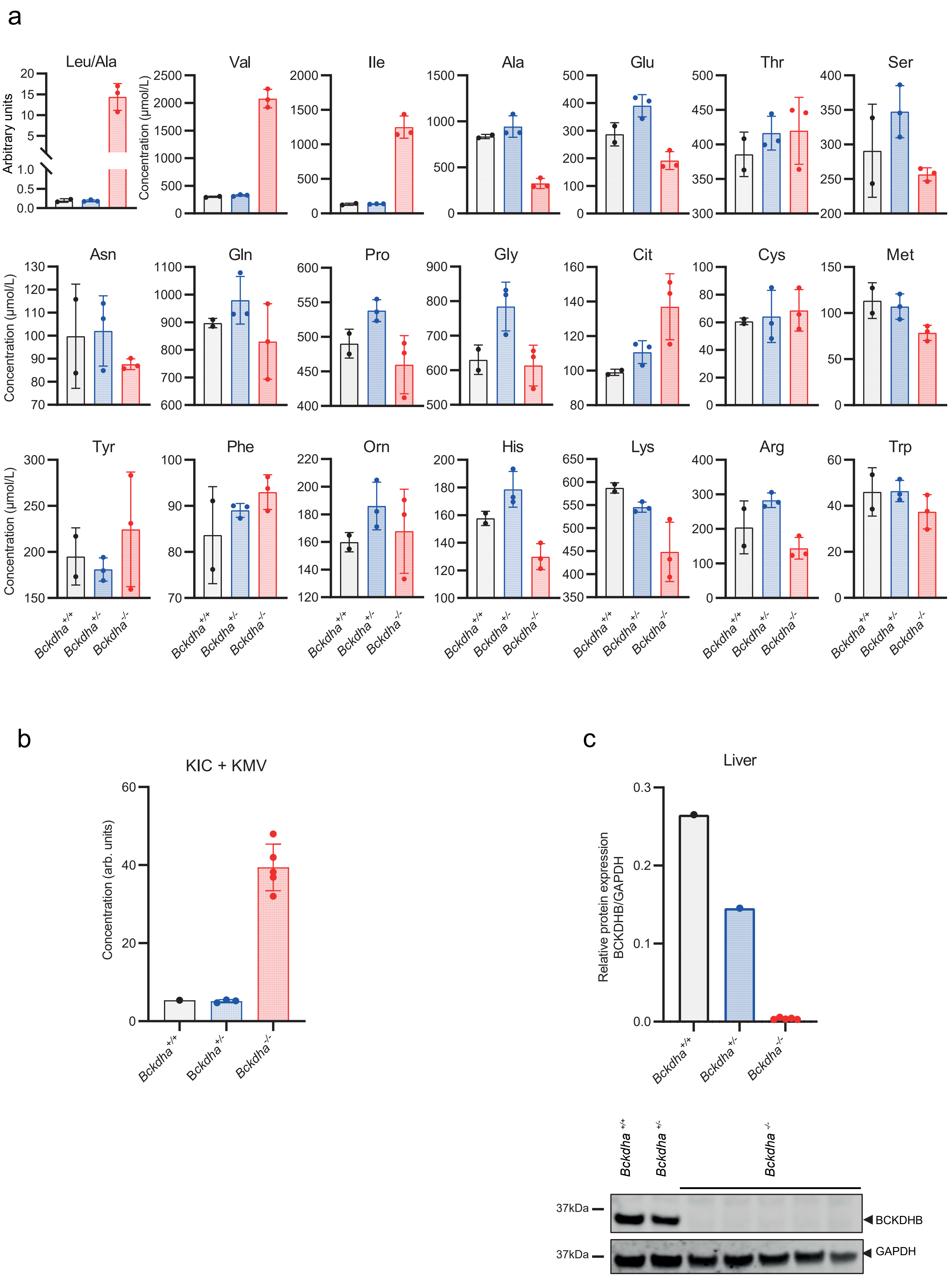

Fig. S1. Additional characterization of Bckdha$^{-/-}$ mice. (a) Amino acid plasma concentrations and leucine/alanine ratio (3 days, *Bckdha*$^{-/-}$ n=3, *Bckdha*$^{+/-}$ n=3, *Bckdha*$^{+/+}$ n=2). (b) Sum of 2-keto-3-methylvaleric (KMV) and 2-ketoisocaproic (KIC) acid plasma concentrations (3 days, *Bckdha*$^{-/-}$ n=5, *Bckdha*$^{+/-}$ n=3, *Bckdha*$^{+/+}$ n=1). (c) BCKDHB protein levels in the liver (1 week, *Bckdha*$^{-/-}$ n=5, *Bckdha*$^{+/-}$ n=1, *Bckdha*$^{+/+}$ n=1). Histograms represent quantification of band intensity normalized to GAPDH levels; data are means ± SD. Source data are provided as Source Data file.

Figure S2

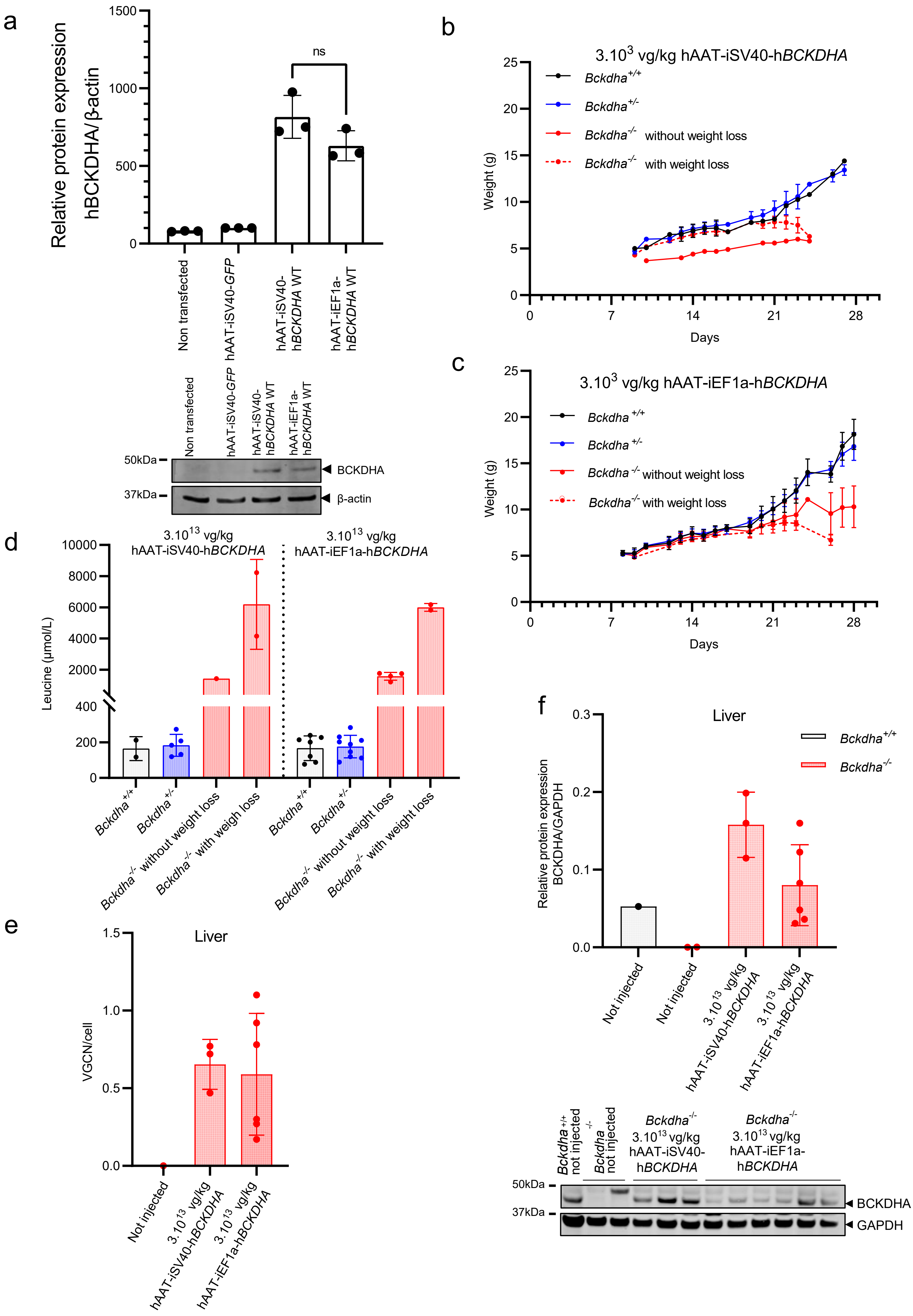

Fig. S2. Comparison of iSV40 and iEFF1α introns in hAAT-h*BCKDHA* constructs. (a) Western blot analyses of human BCKDHA in non-transfected and Huh 7 cells transfected with hAAT-*GFP*, hAAT-iSV40 h*BCKDHA* (construction carrying the hAAT promoter and the SV40 intron) and hAAT-iEF1α-h*BCKDHA* (construction carrying the hAAT promoter and the EF1α intron). Histograms represent quantification of band intensity normalized to actin levels; analyses were performed in triplicate. Two-sided Mann-Whitney test for comparison of hAAT-iSV40 h*BCKDHA* and hAAT-iEF1α-h*BCKDHA* (p-value = 0.2). (b) Weight curves for mice injected at 3 10^{13} vg/kg with AAV8-hAAT-iSV40-h*BCKDHA* (*Bckdha*$^{-/-}$ with weight loss n=2, *Bckdha*$^{-/-}$ without weight loss n=1, *Bckdha*$^{+/-}$ n=5, *Bckdha*$^{+/+}$ n=2) and (c) at 3 10^{13} vg/kg with AAV8-hAAT-iEF1α-h*BCKDHA* (*Bckdha*$^{-/-}$ with weight loss n=2, *Bckdha*$^{-/-}$ without weight loss n=4, *Bckdha*$^{+/-}$ n=9, *Bckdha*$^{+/+}$ n=7). (d) Leucine concentrations in plasma at days 28 or at sacrifice (3 10^{13} vg/kg with AAV8-hAAT-iSV40-h*BCKDHA*: *Bckdha*$^{-/-}$ with weight loss n=2, *Bckdha*$^{-/-}$ without weight loss n=1, *Bckdha*$^{+/-}$ n=5, *Bckdha*$^{+/+}$ n=2; 3 10^{13} vg/kg with AAV8-hAAT-iEF1α-h*BCKDHA*: *Bckdha*$^{-/-}$ with weight loss n=2, *Bckdha*$^{-/-}$ without weight loss n=4, *Bckdha*$^{+/-}$ n=9, *Bckdha*$^{+/+}$ n=7). (e) Vector genome copy number (VGCN) and (f) BCKDHA protein levels in liver (*Bckdha*$^{-/-}$ injected with AAV8-hAAT-iSV40-h*BCKDHA*: n=3 or with AAV8-hAAT-iEF1α-h*BCKDHA*: n=6, controls: *Bckdha*$^{-/-}$ n=1 non injected and sacrificed at 1 week + 1 non injected *Bckdha*$^{+/+}$ sacrificed at 1 months for western blot). The antibody detected both the human BCKDHA and the murine BCKDHA proteins. All data are shown as mean ± SD. Source data are provided as Source Data file.

Figure S3

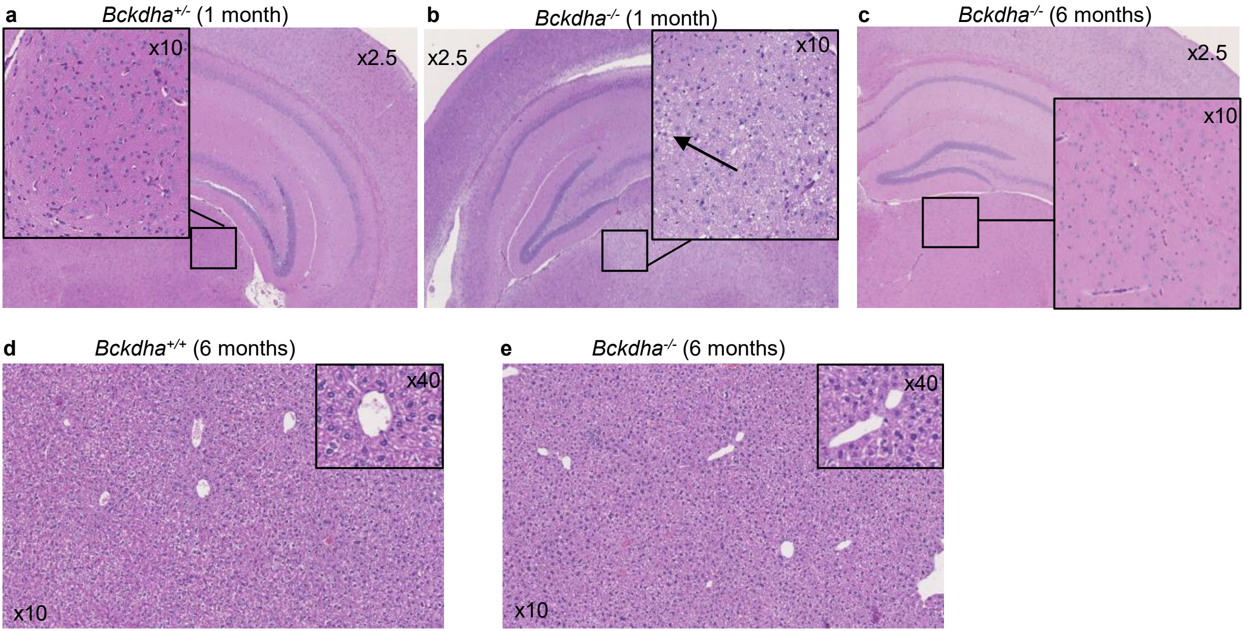

Fig. S3. **Brain and liver pathology of treated *Bckdha*$^{-/-}$ mice**. Representative coronal sections of the brain from *Bckdha*$^{+/-}$ and *Bckdha*$^{-/-}$ mice (n=1 for each), injected at P0 with 10^{13} vg/kg of AAV8-EF1α-h*BCKDHA* vector and sacrificed at 4 weeks (a, b) and of the brain from *Bckdha*$^{-/-}$ mouse, injected at P0 with 10^{14} vg/kg of AAV8-EF1α-h*BCKDHA* vector and sacrificed at 6 months (n=1) (c). Sections were subjected to Hematoxylin-Eosin staining and pictures were acquired using a light microscopy. Diffuse vacuolation was visible in several structures of the *Bckdha*$^{-/-}$ mouse brain injected at 10^{13} vg/kg, including the hippocampus, thalamus, hypothalamus and the midbrain. In contrast, parenchyma of *Bckdha*$^{+/-}$ mouse brain appeared normal. Dense area of vacuolation is presented in the 4-fold enlargement of the thalamus of a *Bckdha*$^{-/-}$ mouse. Enlargement of the same area from a *Bckdha*$^{+/-}$ mouse showed a normal tissue. By comparison, the parenchyma of the *Bckdha*$^{-/-}$ mouse injected at 10^{14} vg/kg appeared normal. Liver sections of *Bckdha*$^{+/+}$ (d) and *Bckdha*$^{-/-}$ (e) mice injected at P0 with 10^{14} vg/kg of AAV8-EF1α-h*BCKDHA* vector and sacrificed at 6 months with normal hepatic parenchyma with Hematoxylin-Eosin staining (x10 magnification) and inlet centred on the centrilobular region (x40 magnification) (n=1 for each).

Figure S4

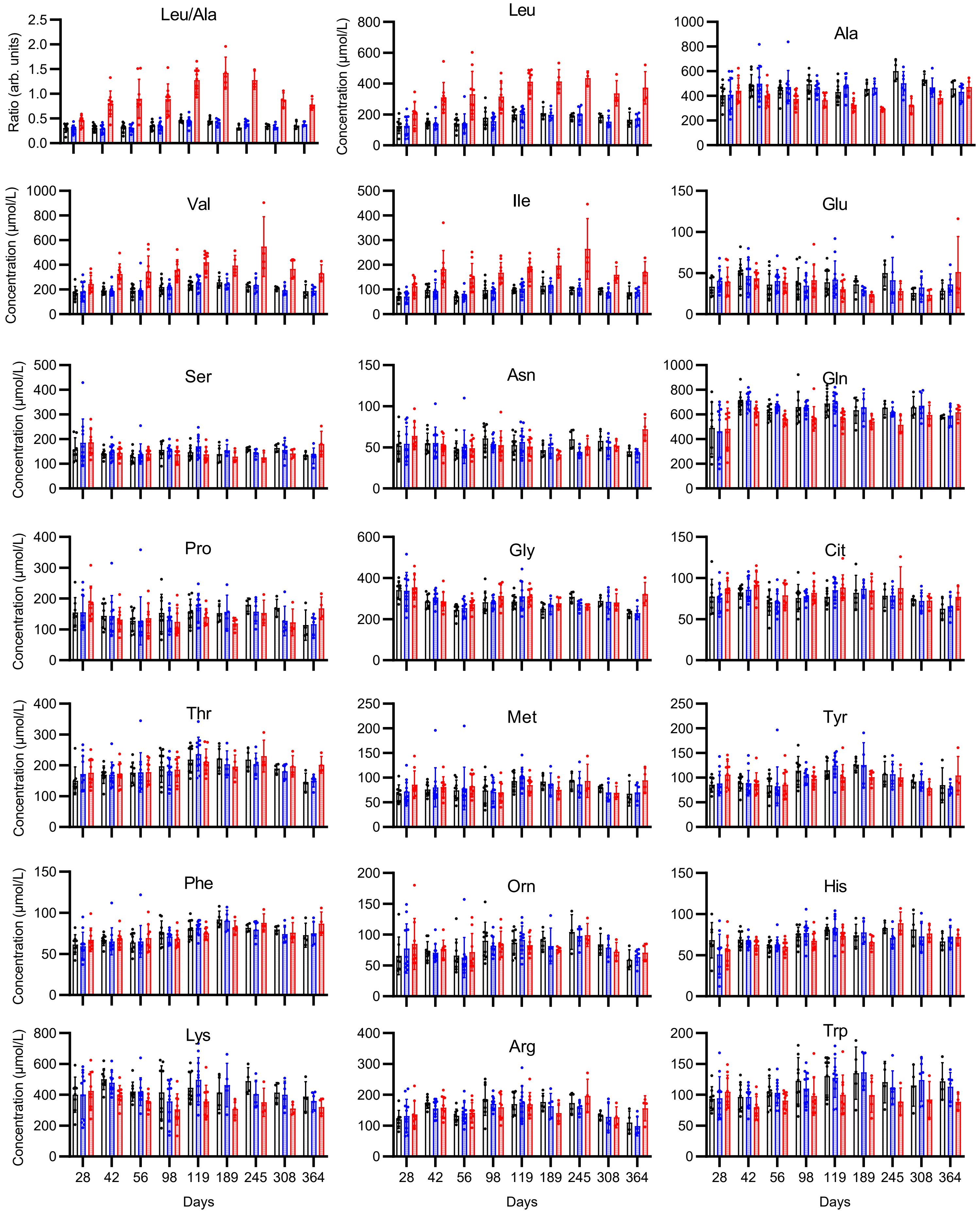

Fig. S4. Longitudinal amino acid analysis in mice treated with 10^{14} vg/kg of AAV8-EF1α-h*BCKDHA*. Amino acid plasma concentrations and leucine/alanine ratio (until 6 months: *Bckdha*$^{-/-}$ n=9, *Bckdha*$^{+/-}$ n=11, *Bckdha*$^{+/+}$ n=9; after 6 months: *Bckdha*$^{-/-}$ n=4, *Bckdha*$^{+/-}$ n=6, *Bckdha*$^{+/+}$ n=4). All data are shown as means ± SD. Source data are provided as Source Data file.

Figure S5

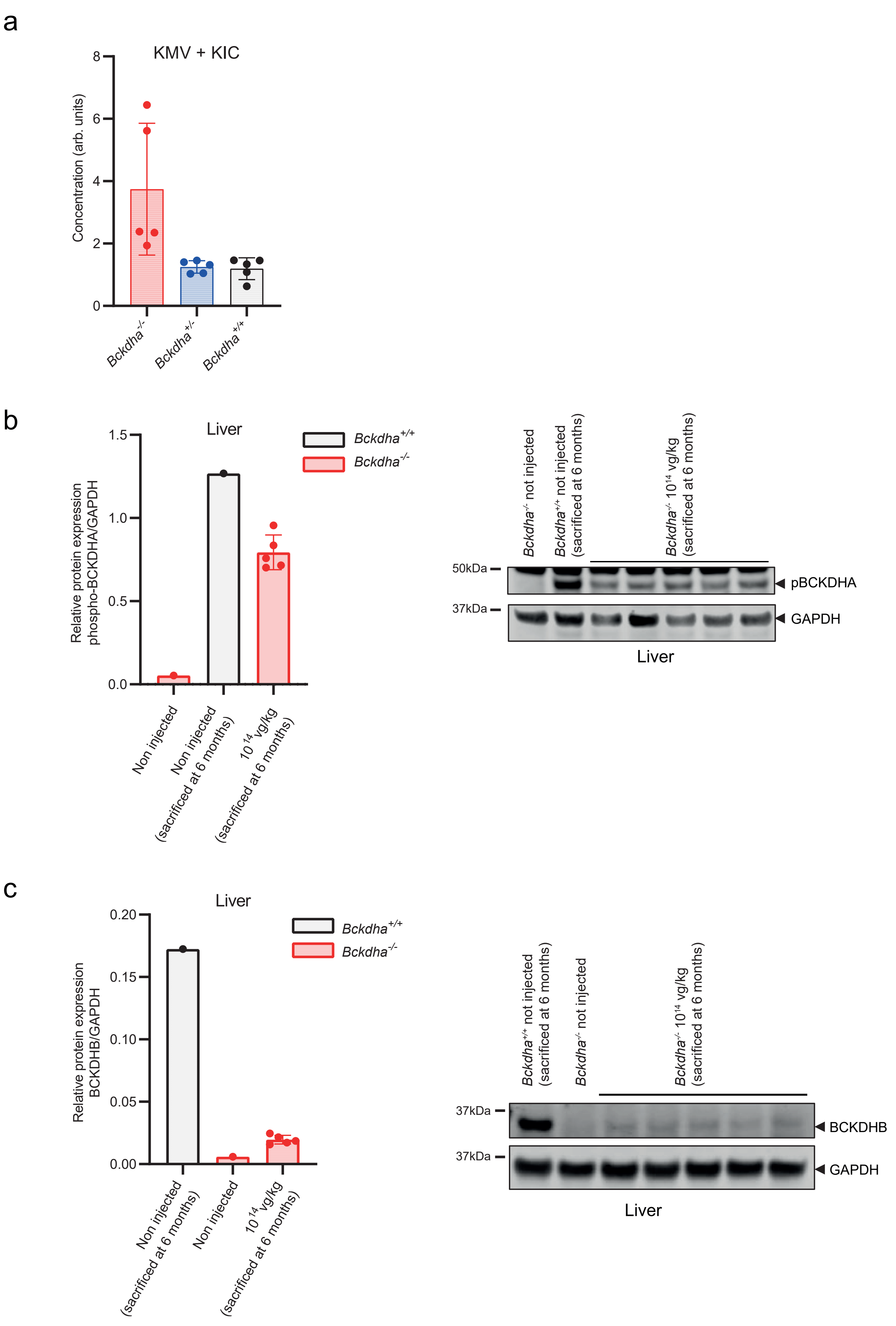

Fig. S5. Additional characterization of *Bckdha*$^{-/-}$ mice injected with 10^{14} vg/kg of AAV8-EF1α-h*BCKDHA* vector and sacrificed at 6 months. (a) Sum of 2-keto-3-methylvaleric (KMV) and 2-ketoisocaproic (KIC) acid plasma concentrations (*Bckdha*$^{-/-}$ n=5, *Bckdha*$^{+/-}$ n=5, *Bckdha*$^{+/+}$ n=5, sacrificed at 6 months, all injected mice). (b) phosphorylated BCKDHA (pBCKDHA) and (c) BCKDHB protein levels in liver in mice (*Bckdha*$^{-/-}$ injected n=5, controls: *Bckdha*$^{-/-}$ n=1 non injected and sacrificed at 1 week + 1 non injected *Bckdha*$^{+/+}$ sacrificed at 6 months). All data are shown as means ± SD. Source data are provided as Source Data file.

Figure S6

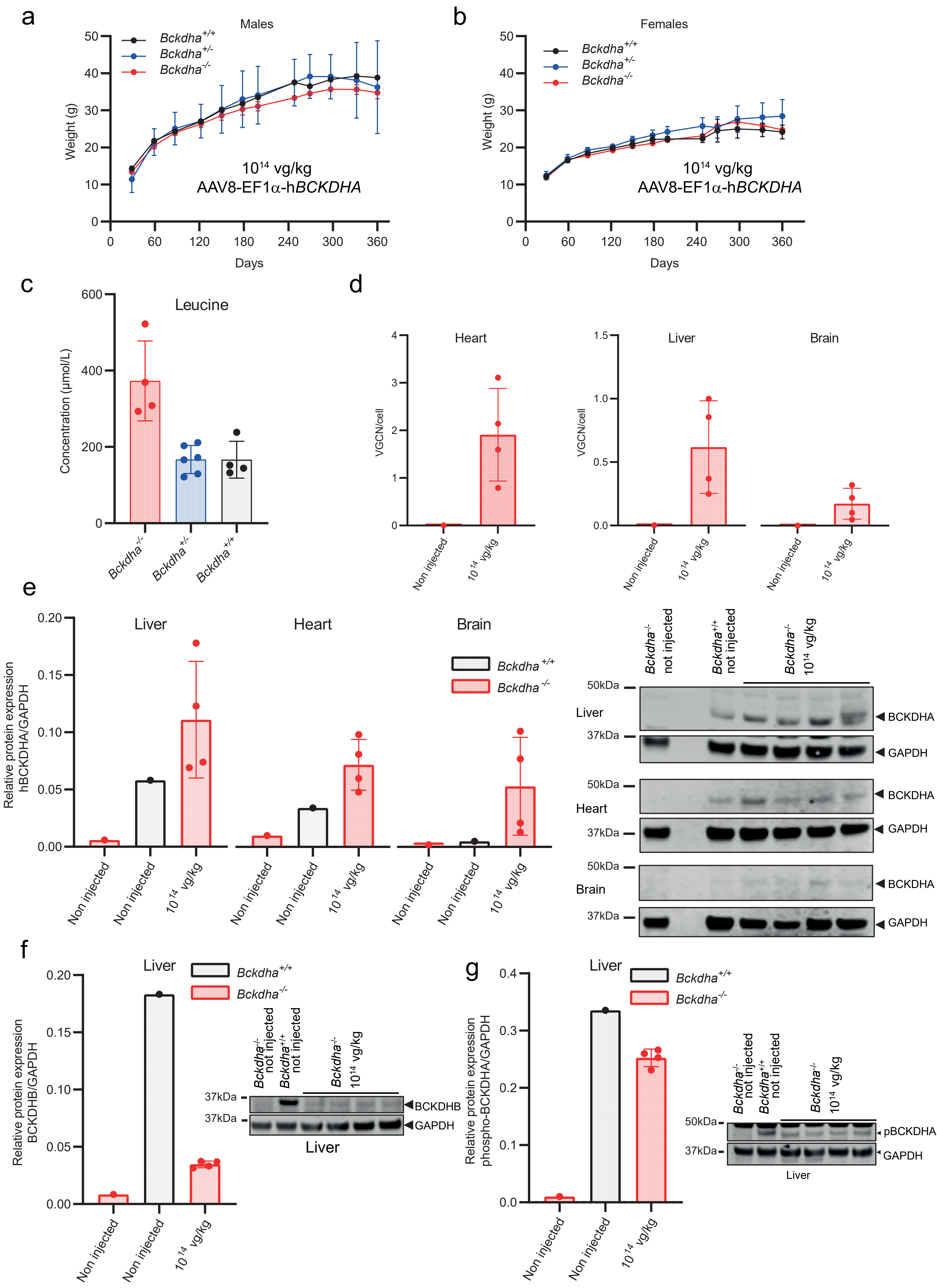

Fig. S6. Additional characterization of *Bckdha*$^{-/-}$ mice injected with 10^{14} vg/kg of AAV8-EF1α-h*BCKDHA* vector and sacrificed at 12 months. (a) Weight curves for males and sacrificed at 12 months (*Bckdha*$^{-/-}$ n=3, *Bckdha*$^{+/-}$ n=2, *Bckdha*$^{+/+}$ n=1) and (b) females (*Bckdha*$^{-/-}$ n=1, *Bckdha*$^{+/-}$ n=4 *Bckdha*$^{+/+}$ n=3). (c) Leucine concentrations in plasma at 12 months (*Bckdha*$^{-/-}$ n=4, *Bckdha*$^{+/-}$, n=6, *Bckdha*$^{+/+}$ n=4, all injected). (d) Vector genome copy number (VGCN) and (e) BCKDHA protein levels in liver, heart and brain in mice sacrificed 12 months after neonatal injection (*Bckdha*$^{-/-}$ injected n=4, controls: *Bckdha*$^{-/-}$ n=1 non injected and sacrificed at 1 week + 1 non injected *Bckdha*$^{+/+}$ sacrificed at 6 months). Analyses were performed in triplicate. The antibody detected both the human BCKDHA and the murine BCKDHA proteins. (f) BCKDHB and (g) phosphorylated BCKDHA (pBCKDHA) protein levels in (*Bckdha*$^{-/-}$ injected n=4, controls: *Bckdha*$^{-/-}$ n=1 non injected and sacrificed at 1 week + 1 non injected *Bckdha*$^{+/+}$ sacrificed at 6 months). All data are shown as means ± SD. Source data are provided as Source Data file.

Figure S7

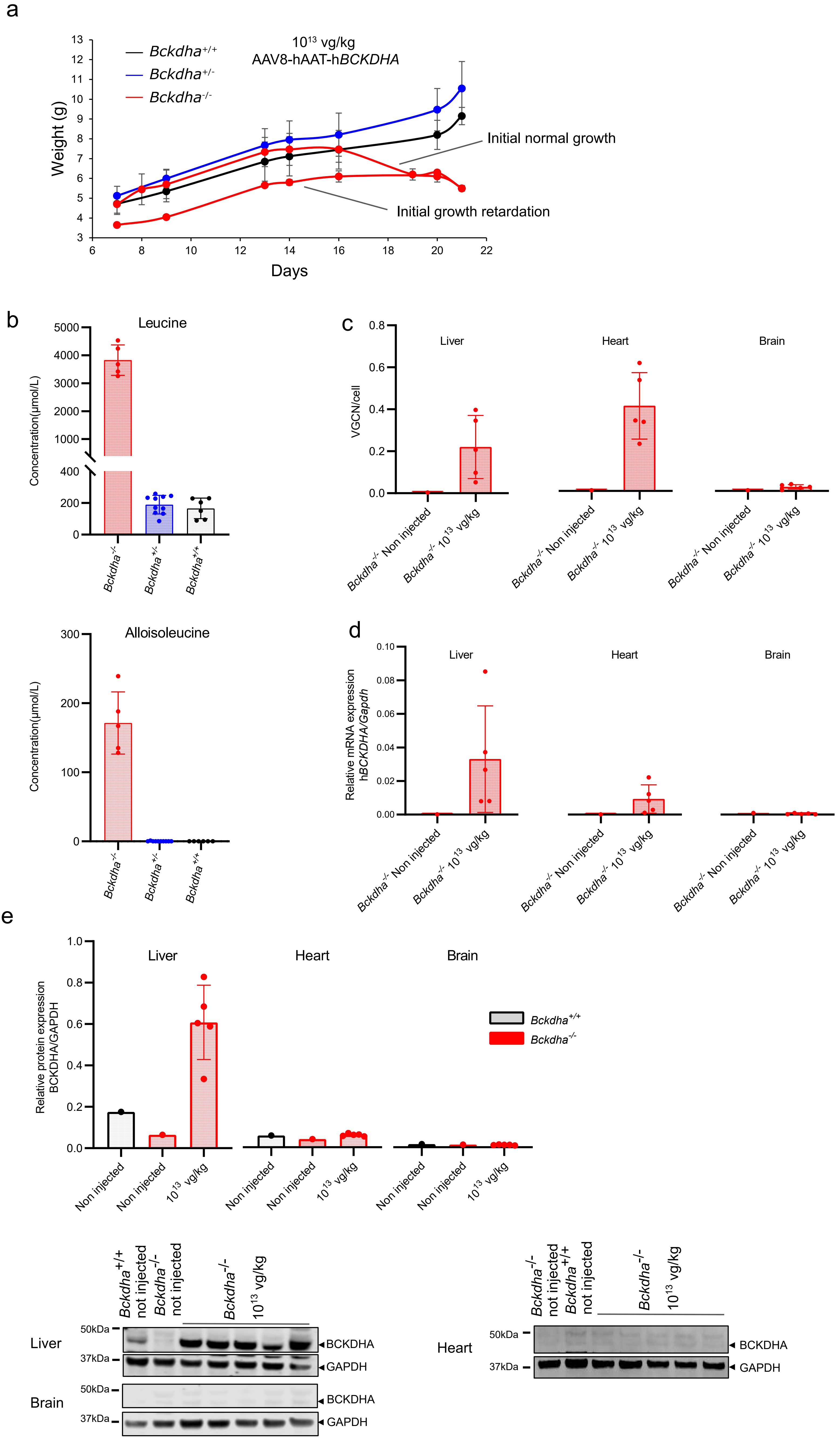

Fig. S7. Liver gene transfer results in transient disease rescue in neonate *Bckdha*$^{-/-}$ mice injected with 10^{13} vg/kg AAV8-hAAT h*BCKDHA* vector. (a) Weight curves and (b) plasma leucine concentrations at sacrifice (19 or 21 days) (*Bckdha*$^{-/-}$ with initial normal growth n=3 (2 males / 1 female) and *Bckdha*$^{-/-}$ with growth on a lower curve n=2 (2 males) (pooled for leucine analysis), *Bckdha*$^{+/-}$ n=10 (4 males / 6 females), *Bckdha*$^{+/+}$ n=6 (3 males / 3 females)). (c) Vector genome copy number (VGCN), (d) human *BCKDHA* (h*BCKDHA*) mRNA and (e) BCKDHA in liver, heart and brain in mice sacrificed 19 or 21 days after neonatal injection (*Bckdha*$^{-/-}$ injected n=5, controls: *Bckdha*$^{-/-}$ n=1 non injected and sacrificed at 1 week + and 1 non injected *Bckdha*$^{+/+}$ mice sacrificed at 4 weeks for western blot); analyses were performed in triplicate. The antibody detected both the human BCKDHA and the murine BCKDHA proteins. All data are shown as mean ± SD. Source data are provided as Source Data file.

Figure S8

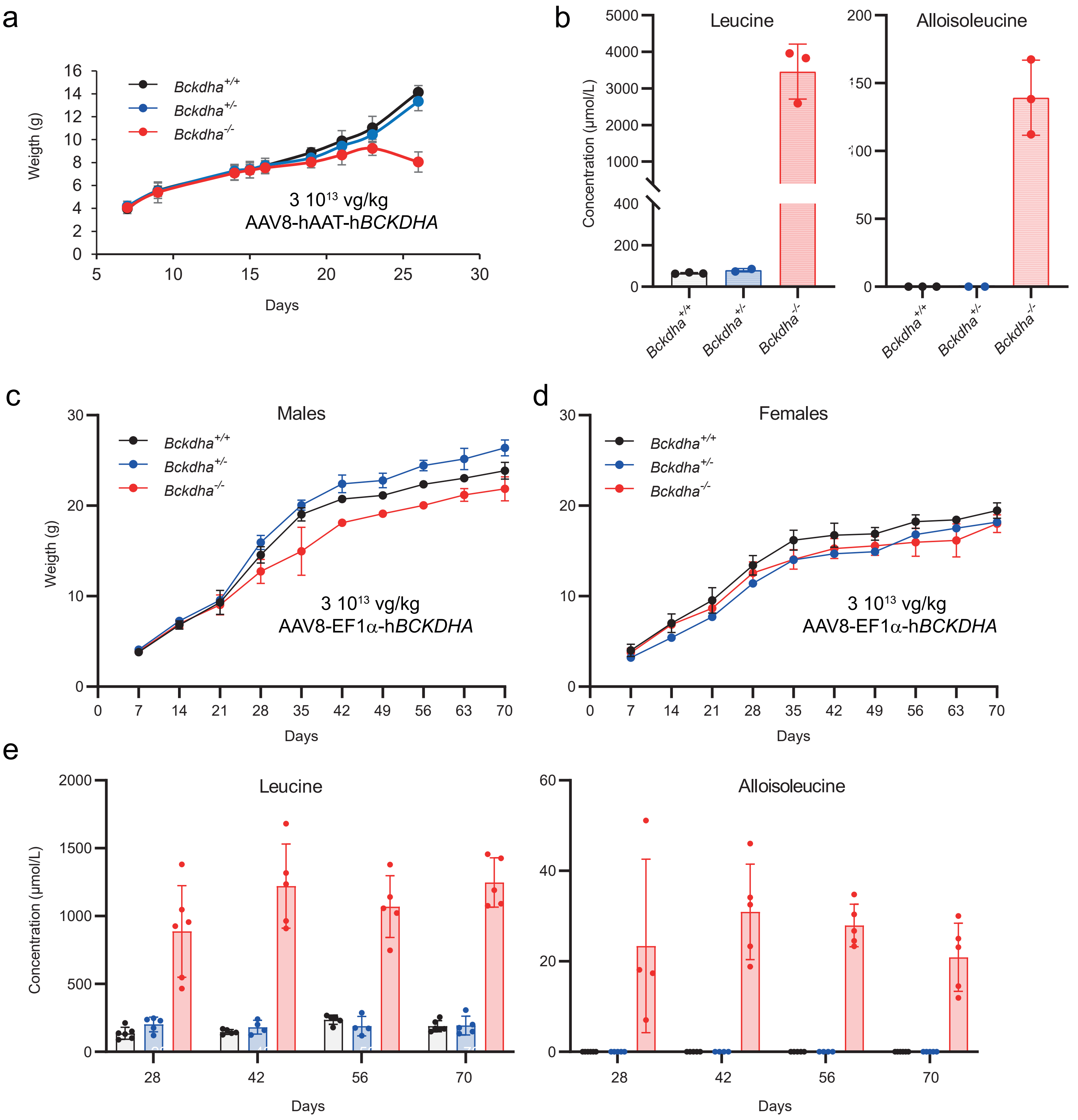

Fig. S8. Comparison of efficacy between AAV8-hAAT-h*BCKDHA* and AAV8-EF1α-h*BCKDHA* at 3 10^{13} vg/kg. Mice were injected at 3 10^{13} vg/kg with AAV8-hAAT-h*BCKDHA*: (a) Weight curves (*Bckdha*$^{-/-}$ n=5 (5 males / 0 females), *Bckdha*$^{+/-}$ n=11 (4 males / 7 females), *Bckdha*$^{+/+}$ n=9 (7 males / 2 females)) and (b) Leucine and alloisoleucine concentrations in plasma at days 28 (*Bckdha*$^{-/-}$ n=3, *Bckdha*$^{+/-}$ n=2, *Bckdha*$^{+/+}$ n=3, all injected). Mice were injected at 3 10^{13} vg/kg with AAV8-EF1α-h*BCKDHA*: (c) Weight curves for males (*Bckdha*$^{-/-}$ n=3, *Bckdha*$^{+/-}$ n=4, *Bckdha*$^{+/+}$ n=3) and (d) females (*Bckdha*$^{-/-}$ n=3, *Bckdha*$^{+/-}$ n=1, *Bckdha*$^{+/+}$ n=3); (e) Leucine and alloisoleucine concentrations in plasma at days 28, 42, 56, 70 (*Bckdha*$^{-/-}$ n=6, *Bckdha*$^{+/-}$ n=5, *Bckdha*$^{+/+}$ n=6, all injected). All data are shown as mean ± SD. Source data are provided as Source Data file.

Figure S9

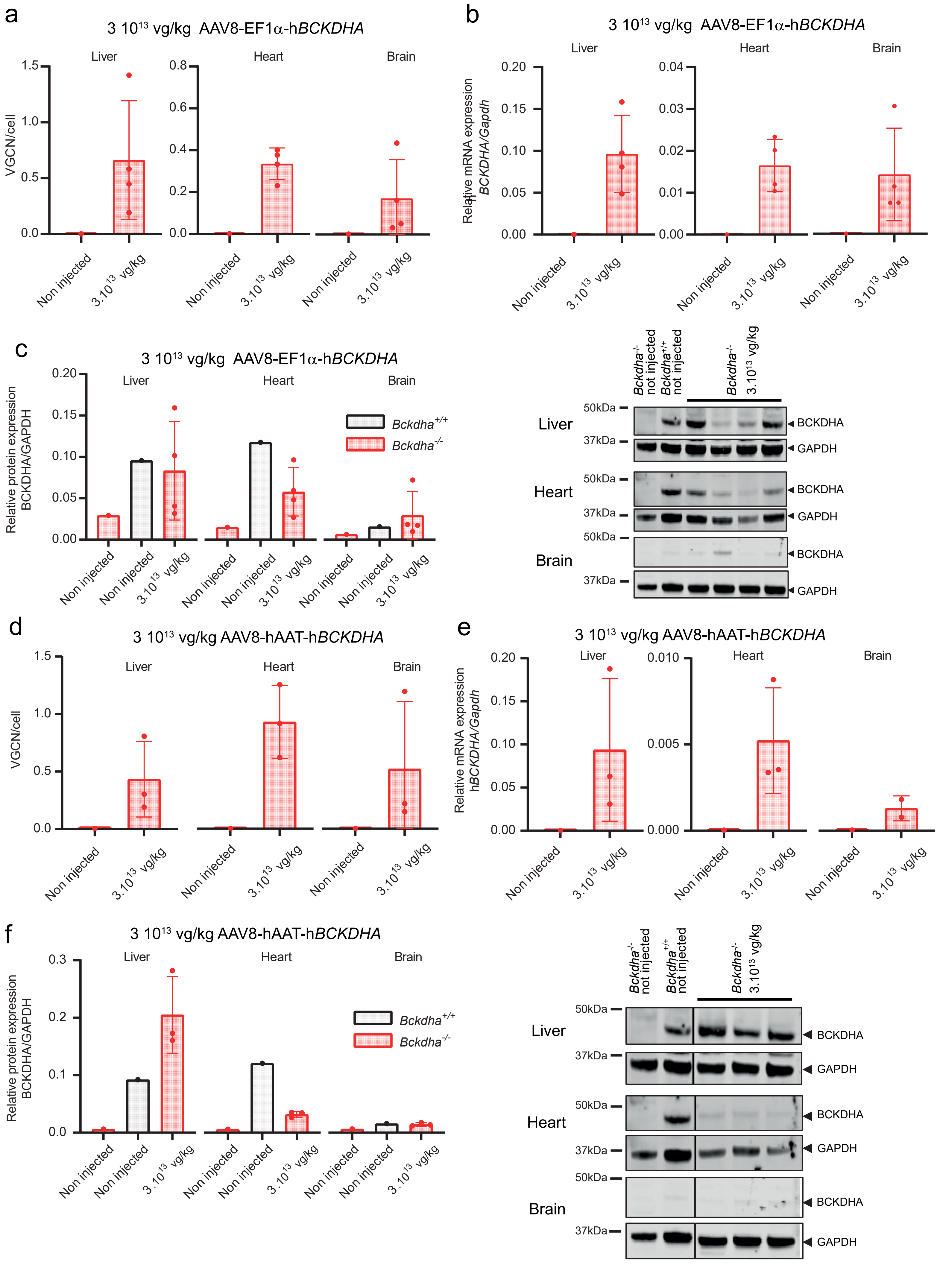

Fig. S9. Additional data regarding comparison of efficacy between AAV8-hAAT-h*BCKDHA* and AAV8-EF1α-h*BCKDHA* at 3 10^{13} vg/kg. Mice were injected at 3 10^{13} vg/kg with AAV8-EF1α-h*BCKDHA*: (a) Vector genome copy number (VGCN), (b) human *BCKDHA* (h*BCKDHA*) mRNA and (c) BCKDHA protein levels in liver, heart and brain in mice at sacrifice (*Bckdha*$^{-/-}$ injected n=4, controls: *Bckdha*$^{-/-}$ n=1 non injected and sacrificed at 1 week + 1 non injected *Bckdha*$^{+/+}$ sacrificed at 1 month for Western blot). Mice were injected at 3 10^{13} vg/kg with AAV8-hAAT-h*BCKDHA*: (d) Vector genome copy number (VGCN), (e) human *BCKDHA* (h*BCKDHA*) mRNA and (f) BCKDHA protein levels in liver, heart and brain in mice at sacrifice (*Bckdha*$^{-/-}$ injected n=3, controls: *Bckdha*$^{-/-}$ n=1 non injected and sacrificed at 1 week + 1 non injected *Bckdha*$^{+/+}$ sacrificed at 1 month for Western blot). The antibody detected both the human BCKDHA and the murine BCKDHA proteins. All data are shown as mean ± SD. Source data are provided as Source Data file.

Figure S10

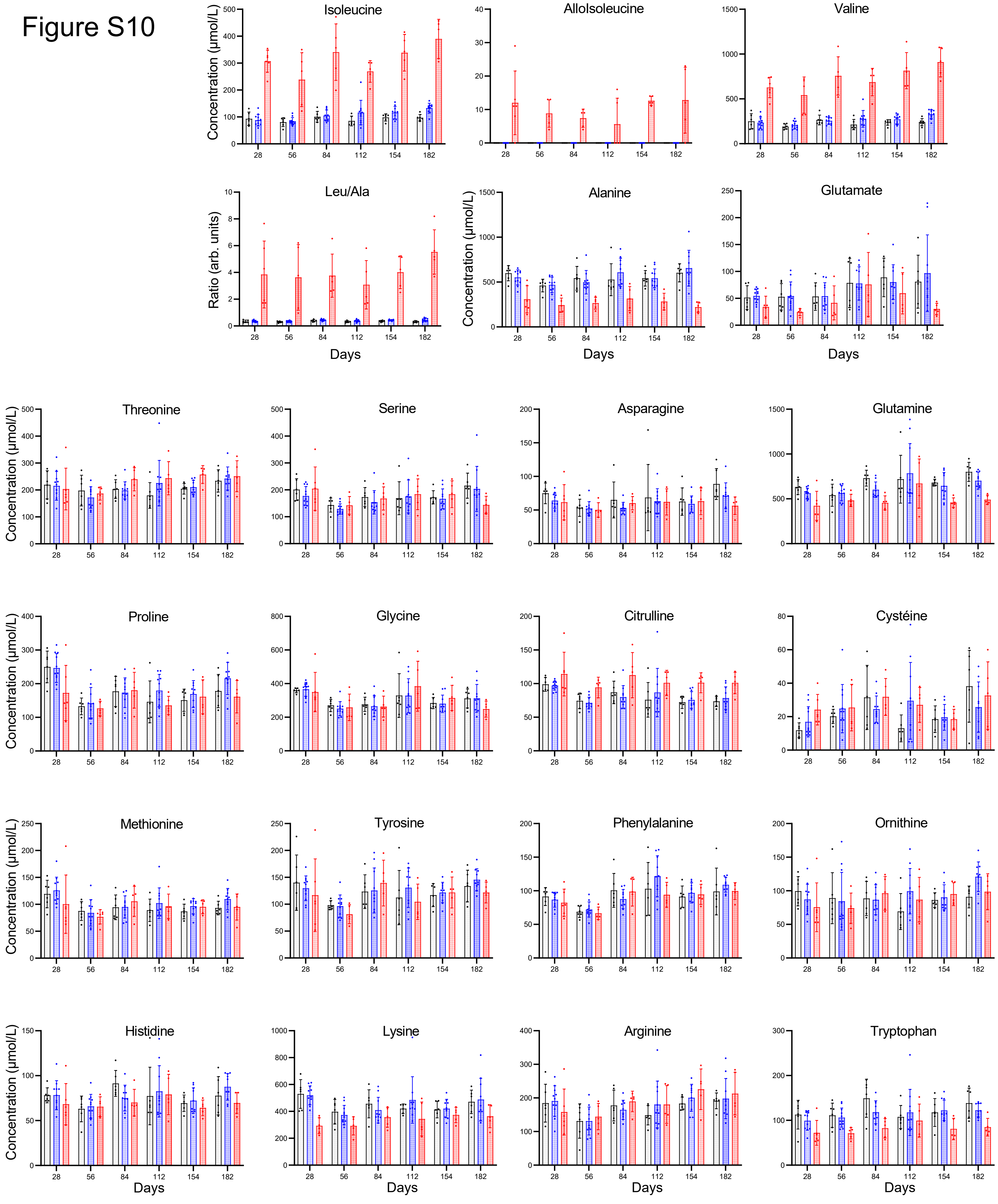

Fig. S10. Longitudinal amino acid analysis in mice treated with 10^{14} vg/kg of AAV8-hAAT-h*BCKDHA*. Amino acid plasma concentrations and leucine/alanine ratio until age 6 months (*Bckdha*$^{-/-}$ n=6, *Bckdha*$^{+/-}$ n=11, *Bckdha*$^{+/+}$ n=6, all injected). One *Bckdha*$^{-/-}$ male mice died at day 28 and was not included for the points > day 28. All data are shown as mean ± SD. Source data are provided as Source Data file.

Figure S11

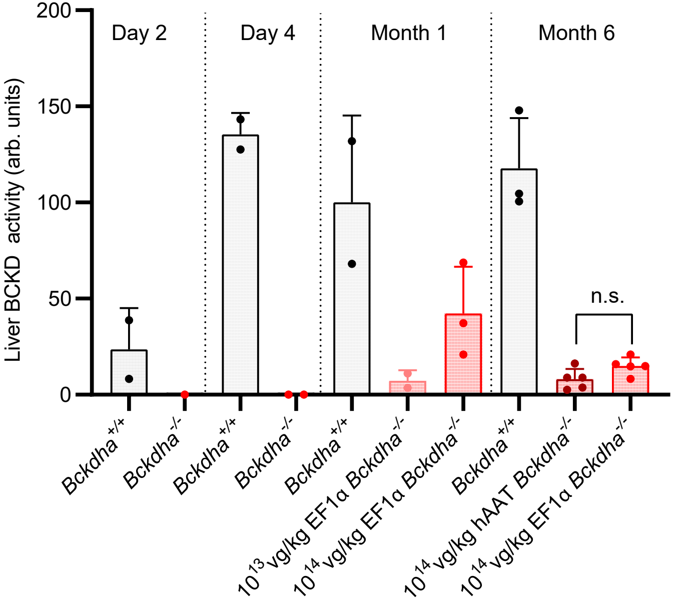

Fig. S11. BCKD enzyme activity in the liver, for untreated mice at 2-4 days (*Bckdha*$^{-/-}$ n=3, *Bckdha*$^{+/+}$ n =4), for *Bckdha*$^{-/-}$ mice treated with the AAV8-EF1α-h*BCKDHA* vector at 10^{13} vg/kg (n=2) and at 10^{14} vg/kg (n=3) and untreated *Bckdha*$^{+/+}$ (n=2), sacrificed at 1 month and for *Bckdha*$^{-/-}$ mice treated with the AAV8-EF1α-h*BCKDHA* vector (n=5) or the AAV8-hAAT-h*BCKDHA* vector (n=5), at 10^{14} vg/kg and untreated *Bckdha*$^{+/+}$ (n=3), sacrificed at age 6 months. Compared to aged-matched controls, at age 6 months, the activity was about 12 ± 4 % and 7 ± 4.5 % in *Bckdha*$^{-/-}$ mice treated respectively with the AAV8-EF1α-h*BCKDHA* or the AAV8-hAAT-h*BCKDHA* vectors at 10^{14} vg/kg (two-sided Mann-Whitney test, p-value=0.222). Values were normalized to protein concentrations and expressed in arbitrary units. All data are shown as mean ± SD. ns: non-significant. Source data are provided as Source Data file.
