# Supplementary material for: Neonatal gene therapy achieves sustained disease rescue of maple syrup urine disease in mice
Source: Nat Commun. 2022 Jun 7;13:3278. doi: 10.1038/s41467-022-30880-w (PMC9174284; doi:10.1038/s41467-022-30880-w)
Supplement: Supplementary file 3 — Reporting Summary [file 41467_2022_30880_MOESM3_ESM.pdf]

nature research

Corresponding author(s): Clément Pontoizeau, Manuel Schiff

Last updated by author(s): Apr 22, 2022

Reporting Summary

Nature Research wishes to improve the reproducibility of the work that we publish. This form provides structure for consistency and transparency in reporting. For further information on Nature Research policies, see our Editorial Policies and the Editorial Policy Checklist.

Statistics

For all statistical analyses, confirm that the following items are present in the figure legend, table legend, main text, or Methods section.

n/a	Confirmed	
☐	☒	The exact sample size (*n*) for each experimental group/condition, given as a discrete number and unit of measurement
☐	☒	A statement on whether measurements were taken from distinct samples or whether the same sample was measured repeatedly
☐	☒	The statistical test(s) used AND whether they are one- or two-sided<br>*Only common tests should be described solely by name; describe more complex techniques in the Methods section.*
☐	☒	A description of all covariates tested
☒	☐	A description of any assumptions or corrections, such as tests of normality and adjustment for multiple comparisons
☐	☒	A full description of the statistical parameters including central tendency (e.g. means) or other basic estimates (e.g. regression coefficient) AND variation (e.g. standard deviation) or associated estimates of uncertainty (e.g. confidence intervals)
☐	☒	For null hypothesis testing, the test statistic (e.g. F, t, r) with confidence intervals, effect sizes, degrees of freedom and P value noted<br>*Give P values as exact values whenever suitable.*
☒	☐	For Bayesian analysis, information on the choice of priors and Markov chain Monte Carlo settings
☒	☐	For hierarchical and complex designs, identification of the appropriate level for tests and full reporting of outcomes
☒	☐	Estimates of effect sizes (e.g. Cohen's d, Pearson's r), indicating how they were calculated

Our web collection on statistics for biologists contains articles on many of the points above.

Software and code

Policy information about availability of computer code

Data collection	NanoDrop (version 1.6.198, nucleic acids concentration assay), QuantStudio (version 5.2, VGCN and RT-qPCR), Image Studio (version 5.2, western blot), MassLinks (version 4.2, LC-MS/MS), Kinetics (version 3.00, spectrophotometric enzymatic assay), NDP.view2 (version 2.7.41, histology)
Data analysis	GraphPad Prism (version 9.3.1), R software (version 3.4.2 http://www.R-project.org)

For manuscripts utilizing custom algorithms or software that are central to the research but not yet described in published literature, software must be made available to editors and reviewers. We strongly encourage code deposition in a community repository (e.g. GitHub). See the Nature Research guidelines for submitting code & software for further information.

Data

Policy information about availability of data

All manuscripts must include a data availability statement. This statement should provide the following information, where applicable:

- Accession codes, unique identifiers, or web links for publicly available datasets
- A list of figures that have associated raw data
- A description of any restrictions on data availability

All data generated or analysed during this study are included in this published article (and its supplementary information files).

Field-specific reporting

Please select the one below that is the best fit for your research. If you are not sure, read the appropriate sections before making your selection.

☒ Life sciences ☐ Behavioural & social sciences ☐ Ecological, evolutionary & environmental sciences

For a reference copy of the document with all sections, see nature.com/documents/nr-reporting-summary-flat.pdf

Life sciences study design

All studies must disclose on these points even when the disclosure is negative.

Sample size	No statistical methods were used to predetermined sample sizes. Sample sizes were determined from similar studies for other diseases (Puzzo et al., Science Translational Medicine 2017, Chandler et al., Molecular Therapy 2010).
Data exclusions	The only excluded data, as defined on pre-established criteria, were early dead individuals in the first hours following AAV injections, as mentioned in the manuscript.
Replication	We performed experimental replicates using a minimum of 3 individuals per condition along with technical replicates for each of the experiments. Therefore, we obtained successful data reproducibility.
Randomization	Randomization was not relevant to our experimental setting as described in the Methods. However, covariates were controlled as much as possible: reduced number of trained operators performing AAV injections, AAV batches prepared by a single person and reproducible mice housing conditions.
Blinding	No need for blinding in our study. Knowing the knockout status of the mice was a prerequisite of our experimental protocol. Histopathology and biochemistry (plasma amino acids) analyses were blinded.

Reporting for specific materials, systems and methods

We require information from authors about some types of materials, experimental systems and methods used in many studies. Here, indicate whether each material, system or method listed is relevant to your study. If you are not sure if a list item applies to your research, read the appropriate section before selecting a response.

Materials & experimental systems

n/a	Involved in the study	
☐	☒	Antibodies
☐	☒	Eukaryotic cell lines
☒	☐	Palaeontology and archaeology
☐	☒	Animals and other organisms
☒	☐	Human research participants
☒	☐	Clinical data
☒	☐	Dual use research of concern

Methods

n/a	Involved in the study	
☒	☐	ChIP-seq
☒	☐	Flow cytometry
☒	☐	MRI-based neuroimaging

Antibodies

Antibodies used	Primary anti-BCKDHA (Rabbit polyclonal ; Abcam ; cat. no. ab126173 ; lot GR126952 ; WB 1:1.000)<br>Primary anti-BCKDHA phospho S293 (Rabbit polyclonal ; Abcam ; cat. no. ab200577 ; lot GR369441 ; WB 1:2.000)<br>Primary anti-BCKDHB (Rabbit polyclonal ; Abcam ; cat. no. ab201225 ; lot GR3244895 ; WB 1:200)<br>Primary anti-GAPDH (Mouse monoclonal ; Clone 6C5 ; Abcam ; cat. no. ab8245 ; lot GR3317834 ; WB 1:1.000)<br>Primary anti-β-actin (Mouse polyclonal ; ProteinTech ; cat. no. 20536-I-AP ; WB 1:1.000)<br>Secondary anti-IgG Rabbit (Donkey polyclonal ; LiCor ; cat. no. 926 32213 ; lot C70918 ; WB 1:10.000)<br>Secondary anti-IgG Mouse (Donkey polyclonal ; LiCor ; cat. no. 926 68072 ; lot D00226 ; WB 1:10.000)
Validation	Anti-BCKDHA : Protocol validated for western blot by testing multiple dilutions of the antibody in human cell line (Huh7) and mouse tissues (see also manufacturer's web site).<br>Anti-BCKDHA phospho S293 : Protocol validated by testing multiple dilutions of the antibody in mouse tissues (see also manufacturer's web site).<br>Anti-BCKDHB : Protocol validated by testing multiple dilutions of the antibody in mouse tissues (see also manufacturer's web site).<br>Anti-GAPDH : Protocol validated for western blot by testing multiple dilutions of the antibody in human cell line (Huh7) and mouse tissues (see also manufacturer's web site).<br>Anti-β-actin : Protocol validated for western blot by testing multiple dilutions of the antibody in human cell line (Huh7) (see also manufacturer's web site).

Eukaryotic cell lines

Policy information about cell lines

Cell line source(s)	Huh7 cell line (ATCC-USA) is a kind gift from Pr. Jessica Zucman-Rossi from Centre de Recherche des Cordeliers - Inserm UMR S1138, Paris, France.<br>HEK293 cell line (ATCC-USA) is a kind gift from Dr. Agnès Rötig from Institut Imagine - Inserm UMR 1163, Paris, France.
Authentication	Non authenticated
Mycoplasma contamination	The cell line was tested negative for Mycoplasma
Commonly misidentified lines (See ICLAC register)	No misidentified cell lines.

Animals and other organisms

Policy information about studies involving animals; ARRIVE guidelines recommended for reporting animal research

Laboratory animals	Bckdha+/- mice were purchased from The Canadian Mouse Mutant repository (C57BL / 6N-Bckdhaem1(IMPC)Tcp). These mice carried a 422 266-bp deletion of Chr7 from 25638173 to 25638438 and insAGAGCC at the heterozygous state. Bckdha-/- mice have never been characterized. Both males and females have been used. Mice were treated at birth and followed over 1, 3, 6 or 12 months.
Wild animals	Our study did not involve wild animals.
Field-collected samples	The study did not involved field-collected samples.
Ethics oversight	Mouse studies were performed according to the French and European legislation regarding animal care and experimentation (2010/63/EU) and approved by the local institutional ethical committee (APAFIS#22754-2018092017287553 v3).

Note that full information on the approval of the study protocol must also be provided in the manuscript.
